# Evaluation of Prognostic Factors Associated with Postoperative Complications Following Pulmonary Hydatid Cyst Surgery

**DOI:** 10.2174/1874306402014010016

**Published:** 2020-07-21

**Authors:** Mojtaba Ahmadinejad, Mozaffar Hashemi, Nasim Azizallahi

**Affiliations:** 1 Department of General Surgery, Alborz University of Medical Sciences, Karaj, Iran; 2 Department of General Surgery, Isfahan University of Medical Sciences, Isfahan, Iran; 3 Department of Microbiology, Alborz University of Medical Sciences, Karaj, Iran

**Keywords:** Hydatid cyst, Segmentectomy, Pulmonary, Postoperative complications, ESR, pulmonary

## Abstract

**Background:**

Hydatid cysts are one of the serious complications following echinococcus infection. The liver and the lungs are the most affected organs, respectively. The severity of the disease is associated with the increase in the number and the size of the cysts, cysts rupture, and systemic effects. The aim of this study is to evaluate prognostic factors that are associated with the increased incidence of postoperative complications following pulmonary hydatid cyst surgery.

**Methods:**

Patients referred to Madani hospital from 2014-2018, presenting pulmonary hydatid cysts were included in this study. All the patients were evaluated based on the following parameters: age, gender, location and size of the cysts, rupture status of the cysts (intact or perforated), type of surgical intervention (capitonnage or segmentectomy) and Erythrocyte Sedimentation Rate (ESR). The factors were then compared with postoperative complications. Statistical analysis of the data obtained was conducted using R-software

**Results:**

Of 76 patients enrolled in our study, 52.63% were males and 47.36% were female. Air leak complication was reported in 13.15% of the patients and 3.94% of the patients were presented with pleural effusion. Postoperative complications were significantly associated with the perforated (ruptured) cysts p= 0.001, segmentectomy p= 0.013, giant hydatid cysts p= 0.007 and ESR p= 0.014. However, the side of the lung was not significantly related to postoperative complications.

**Conclusion:**

Our study reports that perforated cysts, increased size, segmentectomy and abnormal ESR are likely to increase postoperative complications following pulmonary hydatid cysts surgery. Prospective studies with perioperative parameters and greater sample size can help to deduce better inferences.

## 1 INTRODUCTION

Hydatid cyst/Hydatid Disease (HD) is one of the most common lung parasitic infections, as a result of echinococcosis. The zoonotic infection is mostly caused by the echinococcus granulosus parasite in the larval stage belonging to the *Taeniidae* family and Echinococcus genus. It is known to affect 1 million people worldwide [1]. The liver and the lungs are the most affected organs. The eggs of the parasite, when acquired from uncooked food, drinking water, host animals, or soil containing the eggs, by means of portal circulation, settle into the liver. Furthermore, through the portal vein and inferior vena cava, they reach the heart and the lungs, eventually. Lymphatic pathways, venal-venous anastomosis, and direct inhalation of the eggs are also the possible routes of the entrance of the eggs into the lungs [2, 3]. Pulmonary hydatid cysts are diagnosed by means of imaging modalities, lab findings and patients’ history [3].

Several variations have been reported in regard to the prevalence of HD in the Middle East [4]. However, in Iran, the pooled prevalence of the infection is reported to be 23.6%, where, southern part, rural area and female gender are more infected [5]. Echinococcus granulosus infects about 92.75% of these cases [6].

The studies have indicated that several cysts-associated factors are likely to increase postoperative complications of the infection. Furthermore, organ-specific and systemic complications are also associated with intact and ruptured cysts, location and the size of the cysts [7]. Surgical intervention is, therefore, required for the removal of cysts. Delay in the surgery may also lead to increased hospitalization costs and duration [8].

The aim of this study is to investigate prognostic factors associated with postoperative complications following pulmonary hydatid cysts surgery.

## 2 METHODS

In this cross-sectional study, patients presenting pulmonary hydatid cyst referred to Madani hospital were included. Patients with bilateral lung cysts, history of previous treatments and those who denied undergoing surgery, were excluded from this study.

All the patients were briefed about the surgical details and written consents were obtained. The study was approved by the Ethical committee of the Madani hospital.

Preoperative corticosteroids were given to the patients to avoid anaphylactic shock. All surgical procedures were conducted under general anesthesia. The patients underwent posterolateral thoracotomy from 5^th^, 6^th^ or 7^th^ intercostal space, depending on the location of the cysts and the cysts were contained with gas and 20% of sodium chloride solution. As per the location, size and number of the cysts, desired surgical procedures were performed.

Based on the data of previous studies [9, 10], the following variables were included in the study: age, gender, the condition of the cysts (intact/rupture or perforated), the size and the location of the cysts, the type of surgical intervention (cystostomy, bronchial closure and capitonnage or segmentectomy), postoperative complications and erythrocyte sedimentation rate. Patients were followed up for a month for postoperative complications such as rupture, air leak, pleural effusion, empyema and hemoptysis

## 3 STATISTICAL ANALYSIS

Since the dependent variable (complication status) in this study was qualitative, contingency tables and mosaic charts were used to describe the relationship of the independent variables with complication status. Risk difference (RD) indices were also used to measure the relationship. RD index refers to the difference in complication rate with the subgroups of independent variables in the study. The reason for using the RD index in this study is a few numbers of reported adverse events in the subgroup of independent variables. Fisher's exact test and two-sample comparison test were also used. The significance level was set at 0.05 and statistical analysis was performed using R-software.

## 4 RESULTS

Of 76 pulmonary hydatid cyst patients included in the study, 40 (52.63%) were male and 36 (47.36%) were female. The mean age of the patients included was 32 ± 12.2 (minimum 8 years and maximum 68 years). The right lobe lower lobe was the most affected area, reported in 55 patients (72.36%).

The most common postoperative complication was air leak, n=10 (13.15%), followed by 3 cases (3.94%) of pleural effusion. No postoperative mortality was reported among these patients.

### 4.1 Rupture Variable

Fisher exact test showed that there was a significant relationship between rupture status and postoperative complications (p-value = 0.001). The incidence of complications for patients with rupture was 33 times higher than for patients without rupture Table **1**.

The RD index was reported to be 0.34, indicating that the prevalence of complications in patients with rupture was 34%, which is significantly higher than in patients without rupture (p-value = 0.016). The mosaic Fig. (**1**) below illustrates these findings.

### 4.2 Surgery Type Variable

There was a significant relationship between the type of surgery and complication status (p-value = 0.006). The odds ratio for this variable cannot be calculated because the frequency of one of the cells is zero Table **2**.

The RD index was 0.17, which indicates that the incidence of complications in patients undergoing segmentectomy was 17% higher than in patients undergoing capitonnage surgery. This difference was reported to be statistically significant (p-value = 0.013). The mosaic Fig. (**2**) below illustrates the outcomes from Fisher’s test.

### 4.3 Size Variable

The results showed that the relationship between size and complication status was significant (p-value <0.001). The chance of complications for patients with rupture was 33 times higher than for patients without rupture Table **3**.

The RD index was found to be 0.31, indicating that the prevalence of complications in patients with cyst greater than 10cm was 31% higher. The difference was also reported to be statistically significant. (p-value = 0.007), as shown in Fig. (**3**).

### 4.4 Lung Side Variable

The LR subgroup was excluded from the Lung side because of its low frequency. Outcomes showed that there was no significant relationship between lung position variable and the complication status (p-value = 0.61).

The RD index was found to be 0.05, indicating that the prevalence of complications in patients with left lung involvement was not significantly different from patients with right lung disease (p-value = 0.3), as shown in Fig. (**4**).

### 4.5 ESR Variable

The results showed that the relationship between ESR status and complication status was significant (p-value = 0.009) Table **4**.

The RD index of ESR and complication status was 0.161, indicating that the prevalence of complications in patients with abnormal ESR was 16% higher than in patients with normal ESR. The difference was reported to be statistically significant (p-value = 0.014) in Fig. (**5**).

## 5 DISCUSSION

Our study investigated the association of postoperative complications with the size and the rupture status of the cysts, along with the type of surgical intervention, side of the lung and erythrocyte sedimentation rate. We reported that the size, rupture of the cysts, the type of the surgery and abnormal ESR levels were significantly associated with increased postoperative complications. Similar to the results from Sadrizadeh, Haghi [3] study, the most common postoperative complication reported in our study was an air leak.

When comparing capitonnage with segmental resection of the cysts, the former is associated with reduced postoperative airway leak and empyema formation and excellent postoperative outcomes [11]. The findings from our study indicate similar outcomes. The significant difference in the postoperative complication was seen between the two surgical procedures; greater in the segmental resection group. However, in an early study, Turna, Yılmaz [12] reported no additional advantage of capitonnage on the postoperative complication and the duration of the hospitalization.

It is also indicated that the large size of the cysts places pressure on surrounding organs such as the heart, esophagus and trachea and is thereby known to be presented with a greater deal of the complications [8]. Moreover, giant hydatid cysts > 10cm provide surgical challenges. Postoperatively, giant cysts may also be associated with a greater incidence of complications [13], such as air leak [14] and empyema [15]. The sample size of these studies varies massively from that of ours. Furthermore, the data in our study have not been presented with respect to the age group; nonetheless, we report that giant hydatid cysts can significantly lead to increased postoperative complications.

Onal and Demir [7] reported that perforated hydatid cysts lead to increased risk of postoperative complications such as atelectasis, pneumothorax, and broncho-pleural fistula, as compared to intact cysts. Our study reported similar findings; however, most common of all the complications were air leak and pleural effusion.

An increased erythrocyte sedimentation rate is commonly reported in patients presenting HD [16]. Salama, Othman [17] reported that an elevation in ESR along with eosinophilia can be the indicator of multiple-organ involvement and complications such as rupture. Our study presents that increased levels of ESR were significantly correlated with an increased incidence of postoperative complications.

## CONCLUSION

Our study is restricted to some limitations, which include; relatively smaller sample size, compared to other studies, lack of age and gender-based analysis and the use of the single dependent variable, *i.e*., postoperative complications.

The study concludes that advancement in the pathogenesis of the infection, characterized by increased cysts size and perforation of cysts, can lead to surgical challenges and increased incidence of postoperative complications.

Furthermore, segmentectomy can lead to an increased risk of these complications. Patients suspected of the infection are required to undergo an immediate diagnosis and treatment plan in order to reduce systemic effects and surgical adverse.

## Figures and Tables

**Fig. (1):**
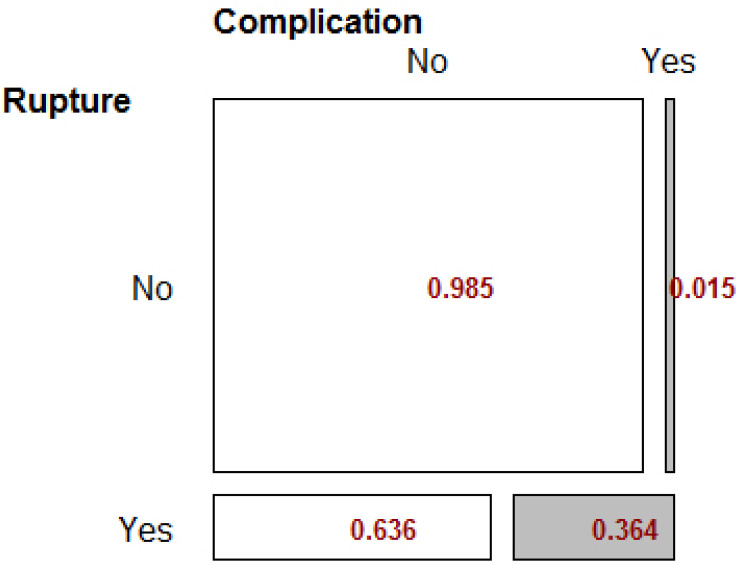
Fisher’s Exact Test for Rupture variable.

**Fig. (2):**
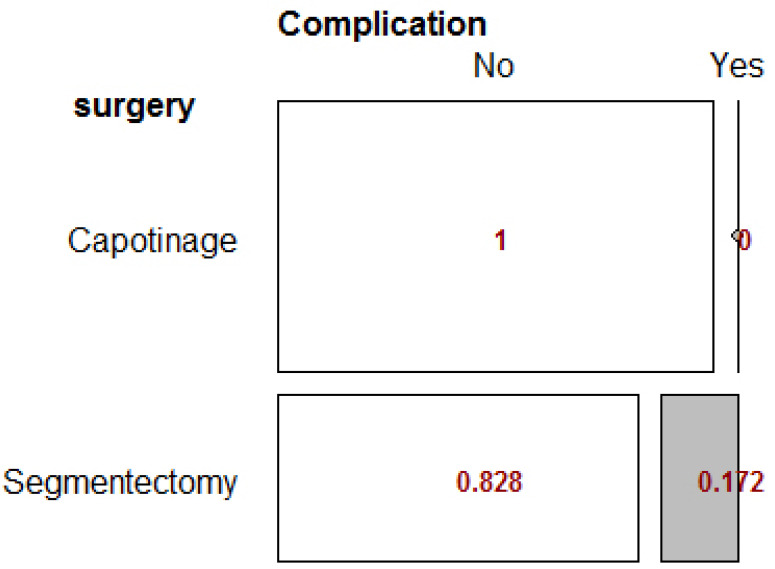
Fisher’s Exact Test for surgery type variables.

**Fig. (3):**
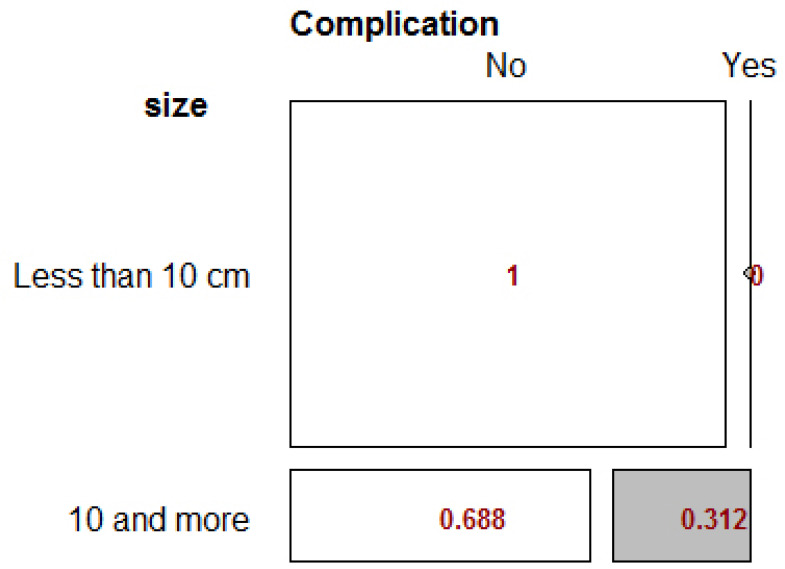
Fisher’s Exact Test for size variable.

**Fig. (4):**
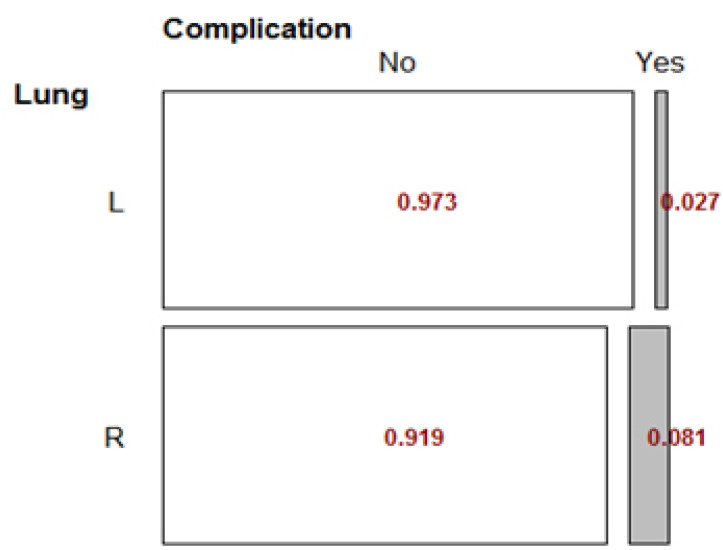
Fisher’s Exact Test for Lung side variable.

**Fig. (5):**
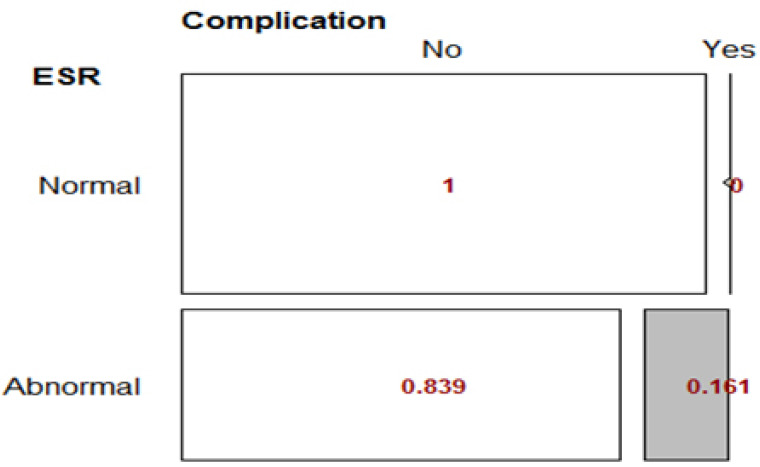
Fisher’s Exact Test for ESR variable.

**Table 1:** The relationship between the variable state of rupture and complications status.

**P value**	**Alternative Hypothesis**	**Odds Ratio**
0.001186 * *	two.sided	33.2

**Table 2:** The relationship between the type of surgery and the complication status.

**P value**	**Alternative Hypothesis**	**Odds Ratio**
0.006428 * *	two.sided	Inf

**Table 3:** Size correlation with complication status.

**P value**	**Alternative Hypothesis**	**Odds Ratio**
0.0002364 * * *	two.sided	Inf

**Table 4:** The relationship between the ESR variable and the complication status.

**P value**	**Alternative Hypothesis**	**Odds Ratio**
0.009197 * *	two.sided	Inf
